# Structural and Functional Insight into ADF/Cofilin from *Trypanosoma brucei*

**DOI:** 10.1371/journal.pone.0053639

**Published:** 2013-01-09

**Authors:** Kun Dai, Shanhui Liao, Jiahai Zhang, Xuecheng Zhang, Xiaoming Tu

**Affiliations:** 1 Hefei National Laboratory for Physical Sciences at Microscale, School of Life Sciences, University of Science and Technology of China, Hefei, Anhui, P.R. China; 2 School of Life Sciences, Anhui University, Hefei, Anhui, P.R. China; Helmholtz Centre for Infection Research, Germany

## Abstract

The ADF/cofilin family has been characterized as a group of actin-binding proteins critical for controlling the assembly of actin within the cells. In this study, the solution structure of the ADF/cofilin from *Trypanosoma brucei* (TbCof) was determined by NMR spectroscopy. TbCof adopts the conserved ADF/cofilin fold with a central β-sheet composed of six β-strands surrounded by five α-helices. Isothermal titration calorimetry experiments denoted a submicromolar affinity between TbCof and G-actin, and the affinity between TbCof and ADP-G-actin was five times higher than that between TbCof and ATP-G-actin at low ionic strength. The results obtained from electron microscopy and actin filament sedimentation assays showed that TbCof depolymerized but did not co-sediment with actin filaments and its ability of F-actin depolymerization was pH independent. Similar to actin, TbCof was distributed throughout the cytoplasm. All our data indicate a structurally and functionally conserved ADF/cofilin from *Trypanosoma brucei*.

## Introduction

As one of the most abundant and conserved cytoskeletal proteins in eukaryotic cells, actin is important for cell migration, intracellular transport, cell division and transcription regulation [1]. Under the control of a large number of actin-binding proteins, the capacity of actin to transit between monomeric (G-actin) and filamentous (F-actin) states is critical for these functions [2], [3].

The actin-depolymerizing factor (ADF)/cofilin family is one of the most important regulators of the spatial and temporal organization of actin filaments. ADF/cofilins induce dissociation of monomers from the pointed ends and sever F-actin to enhance the rate of filament turnover [4]–[7]. Meanwhile, the severing of F-actin leads to an increase of filament assembly by providing new free ends [4], [8], [9]. In addition, recent studies have shown many other roles ADF/cofilins play in phospholipid metabolism, gene regulation and apoptosis cascades [10], [11].

*Trypanosoma brucei* is a eukaryotic unicellular organism, which causes diseases including sleeping sickness in humans and nagana in cattle. Trypanosomes have evolved to adopt several differential forms bearing distinguished strategies to survive in the vector and host. There are two major differential forms during the life cycle of *T. brucei*: the procyclic form in the tsetse fly’s midgut and the bloodstream form in the host. There are many differences between these two forms in cellular procresses such as cell cycle regulation and metabolism [12]. Roles of actin in these two forms are different. Although actin is essential in the bloodstream form of *T. brucei*, depletion of actin in the procyclic-form cells has no influence on the cell growth, except for the distortion and enlargement of the trans region of the Golgi body and the inconspicuous heterogeneous population of vesicles [13]. Furthermore, some studies have demonstrated that the loss of actin has no effect on the export of newly synthesized proteins to the surface of bloodstream and procyclic forms of *T. brucei* [14]. The reasons for actin being essential in the bloodstream but not procyclic forms are not understood. Investigation of the regulation of actin dynamics may provide a clue to answer these questions.

Apotential ADF/cofilin from *T. brucei*, TbCof (Trypanosome Genomic Data Base accession number: Tb927.3.5180) has been reported previously [15]. In this study, we have determined the solution structure of TbCof and characterized its G-actin binding and F-actin depolymerization activities. In addition, immunofluorescence staining indicated that TbCof is localized to the cytoplasm throughout the cell cycle, consistent with its major role in the cytoplasm.

## Results

### Sequence Alignment of ADF/cofilin Family Proteins

**Figure 1:**
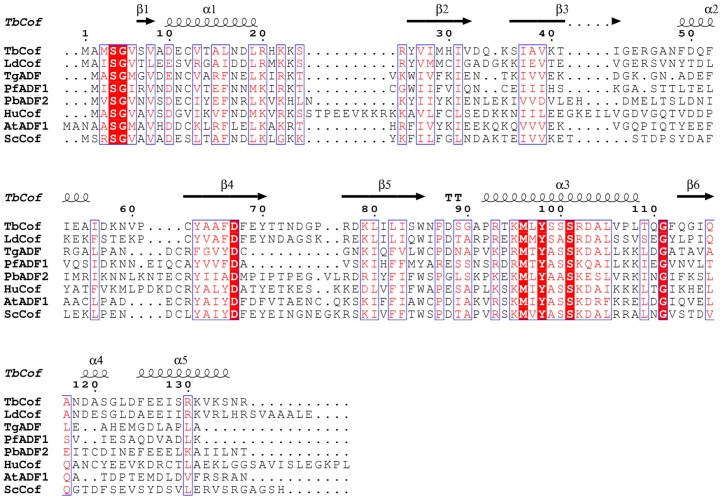
Sequence alignment of TbCof with other ADF/cofilin members. The alignment was prepared using ClustalW2 and ESPript 2.2. Identical residues are boxed in red. The accession numbers of the proteins used for the alignment are listed as follows: Swiss-Prot, Q2QKR1, ADF/cofilin from *Leishmania donovani* (LdCof); Swiss-Prot, B9Q2C8, ADF from *Toxoplasma gondii* (TgADF); Swiss-Prot, P86292, ADF1 from *Plasmodium falciparum* (PfADF1); Swiss-Prot, Q4YT54, ADF2 from *Plasmodium berghei* (PbADF2); Swiss-Prot, P23528, cofilin from *Homo sapiens* (HuCof)*;* Swiss-Prot, Q39250, ADF1 from *Arabidopsis Thaliana* (AtADF1); Swiss-Prot, Q03048, cofilin from *Saccharomyces cerevisiae* (ScCof). The secondary structure elements of TbCof are labeled on the top of the alignment.

A sequence alignment of ADF/cofilin family proteins was performed using ClustalW2 and ESPript 2.2 [16], [17]. The alignment showed that TbCof shares 26%–53% sequence identity with other ADF/cofilin homologs (Fig. 1). Furthermore, the alignment denoted some conserved residues of this protein family. The conserved residues are located mainly in three regions. The first conserved region is within the N-terminus, including S4, G5 and two hydrophobic residues. The second one is comprised of D67 and four hydrophobic residues. The third one includes the residues from R93 to L105, of which M96, Y98 and S101 are conserved in all ADF/cofilin family members.

### Solution Structure of TbCof

**Figure 2:**
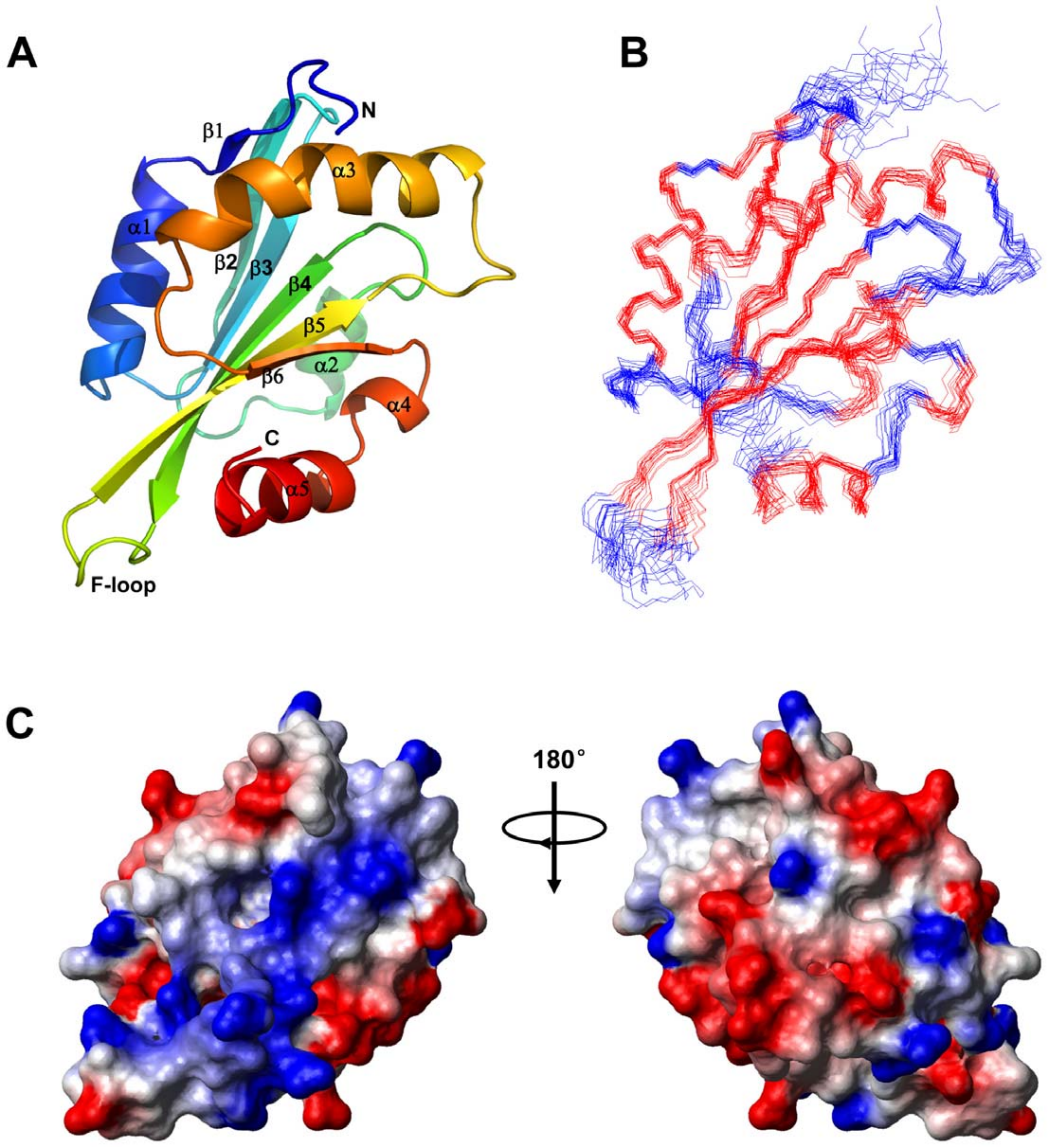
NMR solution structure of TbCof. A. The lowest-energy conformation was used for the cartoon representation of TbCof, showing a central β sheet surrounded by α helices. The key secondary structure elements, both termini, and the F-loop are labeled; B. Superposition of backbone traces of the 20 lowest-energy NMR structures of TbCof. C. Electrostatic surface diagram of the lowest-energy conformation of TbCof is shown from two different orientations 180 degrees apart (red, negative; blue, positive; white, neutral).

The solution structure of TbCof was determined by NMR spectroscopy. Structural parameters for the solution structure of TbCof are summarized in Table 1. The assembly of twenty structures, ribbon representation and electrostatic potential surface of the lowest-energy structure are shown in Fig. 2. In total, 1814 nontrivial NOE distance restraints and 74 hydrogen bond restraints were included in the structure calculation (Table 1). The statistical parameters in Table 1 indicate a high-quality NMR structure of TbCof. The Ramachandran plot [18] shows that 83.9%, 13.3% and 2.8% residues are in the most favored regions, the additionally allowed regions and the generously allowed regions, respectively. No residue is present in the disallowed regions. The backbone RMSD for the secondary structure regions of the assembly of the 20 structures is 0.62 Å. The atomic coordinates for all the 20 structures have been deposited in the Protein Data Bank with the PDB ID code 2LJ8.

**Table 1:** NMR structural statistics.

NMR restraints in the structure calculation	
Intraresidue(i = j)	331
Sequential (|i−j| = 1)	510
Medium-range (|i−j| <5)	381
Long-range (|i−j| >/ = 5)	518
Hydrogen bonds	74
Total distance restraints	1814
Dihedral angle restraints	175
Residual violations	
CYANA target functions, Å	1.29±0.13
NOE upper distance constrain violation	
Maximum, Å	0.16±0.03
Number >0.2 Å	0±0
Dihedral angle constrain violations	
Maximum, °	6.49±0.16
Number >5°	1±0
Vander Waals violations	
Maximum, Å	0.30±0.00
Number >0.2 Å	4±1
Average structural rmsd to the mean coordinates, Å	
Ordered residues^a^, backbone heavy atoms	0.70
Ordered residues^a^, all heavy atoms	1.30
All backbone atoms^b^	0.78±0.15
All heavy atoms^b^	1.33±0.15
PROCHECK G-factors raw score(Φ and Ψ/all dihedral angls)^a^	−0.37/−0.77
PROCHECK G-factors Z-score(Φ and Ψ/all dihedral angls)^a^	−1.14/−4.55
PMOLPROBITY clash score (raw/Z-score) a	23.38/−2.45
Ramachandran plot summary a (%)	
Most favored regions	83.9
Additionally allowed regions	13.3
Generously allowed regions	2.8
Disallowed regions	0.0

a Selected residues: 7–42, 47–74, 76–110, 112–135.

b Obtained for residues 6–136 since no long-range NOEs were identified for the first five amino acids.

The TbCof structure presents a typical ADF/cofilin fold, which is built by a central β-sheet surrounded by α helices. The central β-sheet is composed of six β-strands (β1: residues 7–8, β2: residues 26–32, β3: residues 36–42, β4: residues 62–70, β5: residues 77–85 and β6: residues 113–116). Four central strands (β3-β2-β4-β5) run antiparallel to each other, whereas strands β1-β3 and β5-β6 run parallel to each other. The five main α-helices (α1: residues 10–20, α2: residues 49–55, α3: residues 92–108, α4: residues 119–123 and α5: residues 125–134) flank either side of the central β-sheet, with α1 and α3 located on one face, α2 and the C-terminal end (119–136) on the opposite. Helix α1 is a standard α helix, which is parallel to the strand β3. Helix α2 is a short α helix on the other side of the strand β3. There is a kink in the center of the longest helix α3 at residues S101 and R102. This kink is a conserved structural feature of ADF/cofilins. The C terminus contains two helices: a short helix α4 within the loop between β6 and the C-terminal helix, and helix α5 which is parallel to the strand β6.

### Structural Comparison of TbCof with other ADF/cofilin Members

The structural comparisons between TbCof and other ADF/cofilin members were performed by the DaliLite version 3.0 [19]. DALI search in Protein Data Bank indicated that TbCof shares high structural similarity with other ADF/cofilin family members. The ADF/cofilin from *Leishmania donovani* (PDB ID: 2KVK) [20] and the cofilin from *Saccharomyces cerevisiae* (PDB ID: 1COF) [21] are the ones with the highest structural similarity to TbCof. The Cα RMSD values between TbCof and *Leishmania donovani* ADF/cofilin and between TbCof and *Saccharomyces cerevisiae* cofilin are 2.7 Å and 2.9 Å, with Z-scores of 14.8 and 14.6, respectively. Besides, TbCof shares high structural similarity with vertebrate and plant ADF/cofilins such as coactosin-like protein from *Mus musculus* (PDB ID: 1UDM) [22], cofilin from *Homo sapiens* (PDB ID: 1Q8G) [23] and ADF1 from *Arabidopsis thaliana* (PDB ID: 1F7S) [24]. The RMSD values between TbCof and these ADF/cofilins range from 2.4 Å to 3.3 Å.

**Figure 3:**
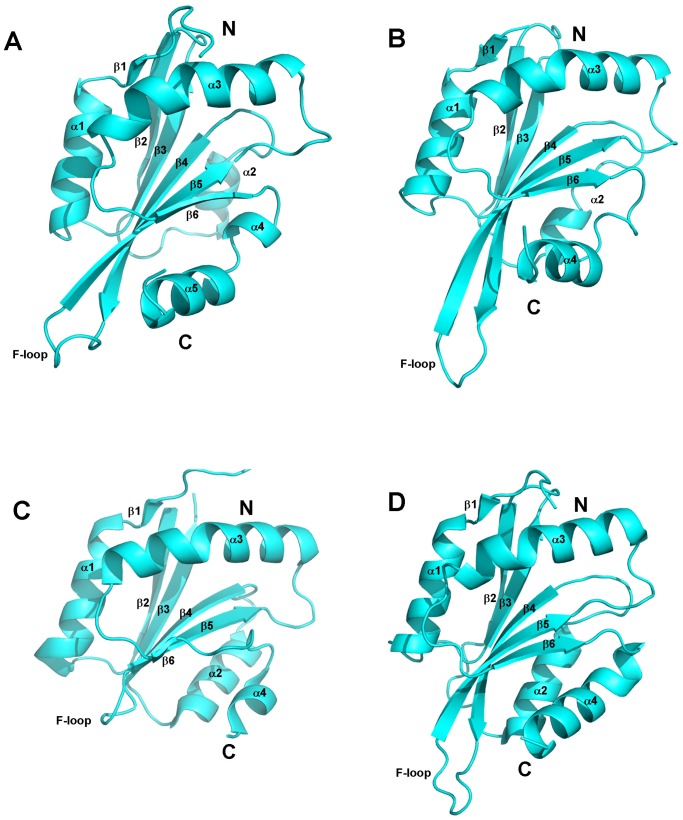
Structural comparison of TbCof with other ADF/cofilin family members. A. ADF/cofilin from *Trypanosoma brucei*; B. cofilin from *Saccharomyces cerevisiae* (PDB ID: 1COF); C. ADF1 from *Plasmodium falciparum* (PfADF1) (PDB ID: 2XF1); D. ADF2 from *Plasmodium berghei* (PbADF2) (PDB ID: 2XFA). These ADF/cofilin family members all share the classical fold except for a short helical turn in the loop between β6 and the C-terminal helix from residue D119 to L123 in TbCof and the shorter C-terminal region in PfADF1. The key secondary structure elements are labeled.

The structures of the cofilin from *Saccharomyces cerevisiae* (PDB ID: 1COF) [21] and the ADFs from *Plasmodium* [25], another unicellular eukaryotic parasite, were compared with that of TbCof (Fig. 3). A short helical turn, which is located within the loop between β6 and the C-terminal helix in TbCof, is absent in yeast cofilin. There are obvious differences between TbCof and PfADF1 in the loop between β4-β5 and the C-terminal helices. In addition, the C-terminal region is relatively shorter in PfADF1. Despite the above differences, all the structures of these ADF/cofilin family members share the classical fold. The conserved ADF/cofilin fold of TbCof suggests a role for TbCof in actin filament turnover.

### Interactions between TbCof and G-actin

**Figure 4:**
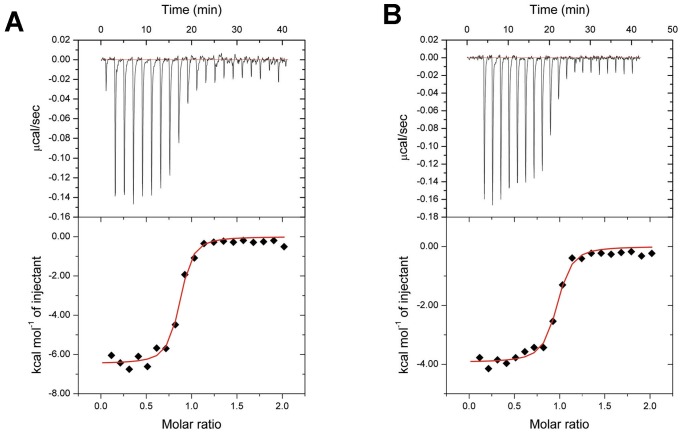
Interactions between TbCof and G-actin revealed by ITC. The negative peaks indicate an exothermic reaction. The area under each peak represents the heat released after an injection of TbCof into G-actin solution (upper panel). Binding isotherms were obtained by plotting the peak areas against the molar ratio of TbCof to G-actin (lower panel). The lines represent the best-fit curves obtained from least-squares regression analyses assuming a one-site binding model. A. ITC of TbCof-G-actin in the presence of ADP. B. ITC of TbCof-G-actin in the presence of ATP.

It has been reported that low ionic strength can increase the actin binding affinity of ADF-H proteins, at least in the twinfilin family and ADF1 from *Arabidopsis thaliana* [9], [26]. To further reveal whether TbCof binds to actin under low ionic conditions, we investigated the interactions between TbCof and G-actin by isothermal titration calorimetry (ITC). The results indicated that TbCof was able to bind to G-actin in the presence of ADP or ATP (Fig. 4A and 4B). ITC titration of TbCof with G-actin revealed a 1∶1 stoichiometry and dissociation constant (K_d_) values of 0.08 and 0.36 µM for ADP-G-actin and ATP-G-actin, respectively, with ΔH of −9.48×10^3^±130.40 cal/mol, ΔS of 10.3 cal/mole/deg, TΔS of 3.01×10^3^ cal/mole, ΔG of −12.50×10^3^ cal/mol for ADP and ΔH of −3.93×10^3^±77.85 cal/mol, ΔS of 17.2 cal/mole/deg, TΔS of 5.04×10^3^ cal/mole, ΔG of −8.97×10^3^ cal/mol for ATP. These results denoted a submicromolar affinity between TbCof and G-actin, and showed that TbCof binds ADP-G-actin with almost five times higher affinity than ATP-G-actin under low ionic conditions.

### Docking of TbCof with G-actin

**Figure 5:**
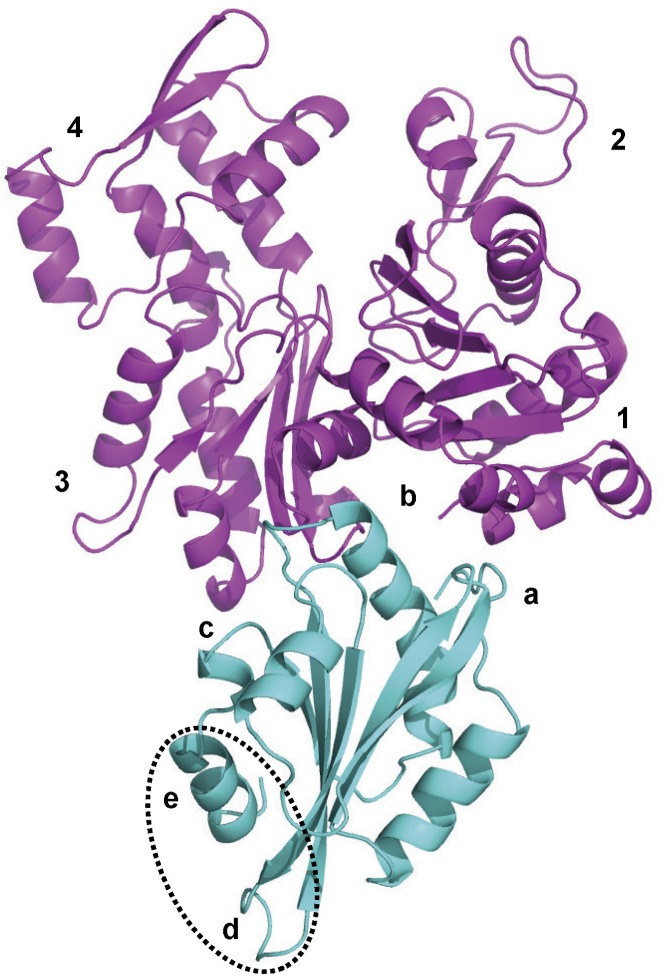
Model of TbCof (cyan) in complex with G-actin (magenta). The G-actin binding site contains 3 regions: a, the N-terminal extension that interacts with actin subdomain 1; b, the long kinked helix α3 that binds to the cleft between actin subdomains 1 and 3; c, the region before the C-terminal α-helix that interacts with actin subdomain 3. F-actin binding is mediated by additional regions consisting of the F-loop between β4-β5 (with label d) and the C-terminal α-helix (with label e). The F-actin binding site is circled by a dashed line.

To identify the residues involved in the interactions between TbCof and G-actin, a model of TbCof in complex with G-actin was generated. Although the sequence identity between TbCof and twinfilins’ C-terminal ADF-H domain (Twf-C) is only 16%, the structures of TbCof and Twf-C adopt the conserved ADF/cofilin fold with an RMSD value of 2.6 Å for the superposition of 122 C alpha atoms. Therefore, the structure of Twf-C in complex with G-actin [27] was used as a template for building the TbCof/G-actin model using the HADDOCK software [28]. 171 structures, representing 85.5% of the water-refined models HADDOCK generated, were classified into 11 clusters. The top cluster was the most reliable according to HADDOCK. The model of TbCof in complex with G-actin is shown in Fig. 5. The statistics of the top cluster are shown in Table 2.

**Table 2:** Statistics of the model of TbCof in complex with G-actin.

HADDOCK score	−45.7±8.7
Cluster size	30
RMSD from the overall lowest-energy structure	2.6±2.2
Van der Waals energy	−39.4±10.2
Electrostatic energy	−365.7±120.3
Desolvation energy	64.9±21.0
Restraints violation energy	30.18±19.5
Buried Surface Area	1543.8±151.1
Z-Score	−2.4

The results showed that TbCof exhibits a conserved binding surface for G-actin. The typical G-actin binding sites of ADF/cofilins are located mainly in the N-terminal unstructured region, the long kinked α-helix, and the loop just before the C-terminal α-helix [23], [27], [29]. The three G-actin binding regions are all present in the TbCof-G-actin model. At the N-terminus of TbCof, residues M1 to S4 are close to residues L346, S348, L349, T351, F352 and F375 from subdomain 1 of G-actin. A similar result was also observed for yeast cofilin, whose first five amino acids are essential for G-actin binding [29]. In the long kinked helix α3 of TbCof, residues R93, K95, M96, L97 and S99 are in close proximity to residues Y143, A144, S145, G342, I345, L346 and S348 from the cleft between the subdomains 1 and 3 of G-actin. Residues R93 and K95 of TbCof (corresponding to R96 and K98 of yeast cofilin) are two highly conserved basic residues that have been implicated in G-actin binding in several ADF/cofilins [23], [29]. In the region before the C-terminal helix, residues G114 and Q116 are in close proximity to residues Y166 and E167 from subdomain 3 of G-actin.

### Depolymerization of Actin Filaments by TbCof

**Figure 6:**
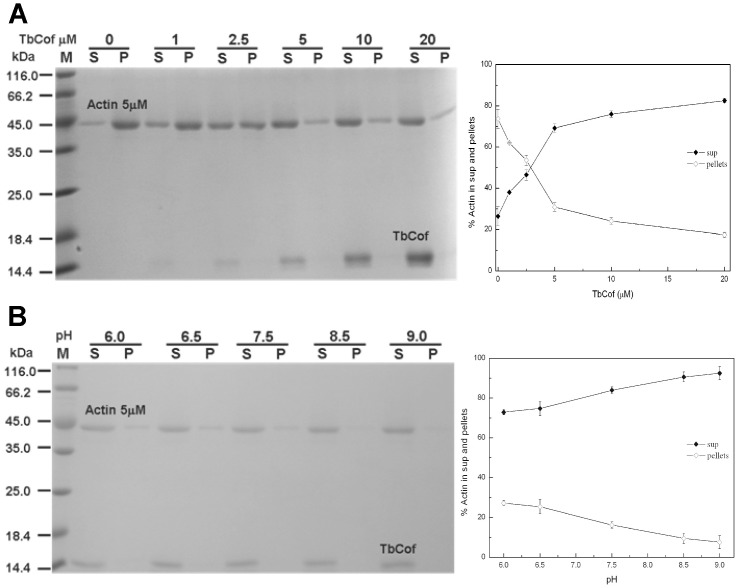
Depolymerization of F-actin by TbCof. A. Left: coomassie blue-stained SDS-PAGE gel of the supernatant (S) and pellet (P) fractions. TbCof does not co-sediment with F-actin at any concentration under the same buffer conditions. Right: quantitative analysis of F-actin depolymerization by TbCof at varying concentrations. B. Effect of pH on F-actin depolymerization and its co-sedimentation with TbCof at an equimolar concentration (5 µM). Left: coomassie blue-stained SDS-PAGE gel of the supernatant (S) and pellet (P) fractions. TbCof depolymerizes F-actin under different pH conditions. Right: quantitative analysis of pH-independent F-actin depolymerization of TbCof.

The effect of TbCof on depolymerization of actin filaments was examined by a co-pelleting assay with preassembled F-actin (Fig. 6). Although TbCof did not co-sediment with F-actin, the amount of unpolymerized actin in the supernatant increased in the presence of TbCof. The results indicate that TbCof primarily depolymerizes actin filaments. Nearly complete depolymerization was observed when a two-fold molar excess or more TbCof was added (Fig. 6A). It has been reported that the interactions between ADF/cofilin and actin are pH-dependent [30], [31]. The analyses of the effects of pH on the actin binding and depolymerization activities of TbCof at a pH range of 6.0–9.0 were performed (Fig. 6B). No change was observed in either actin binding or depolymerization activities of TbCof under any of these conditions. The results demonstrated that the abilities of TbCof to bind to and depolymerize F-actin are pH-insensitive.

**Figure 7:**
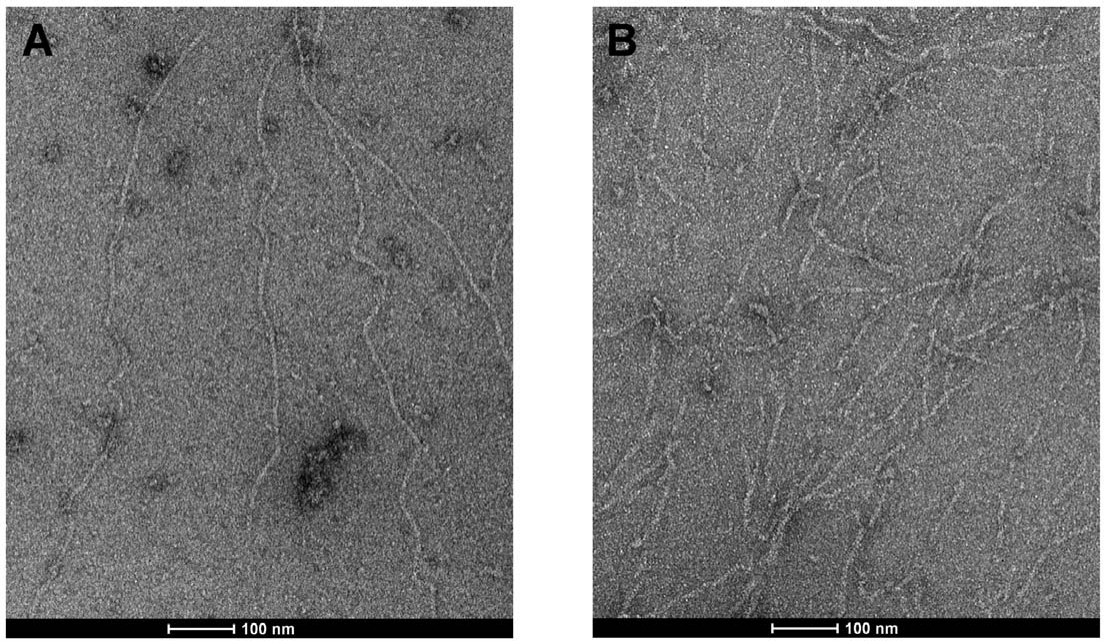
Effect of TbCof on F-actin examined by electron microscopy. F-actin (5 µM) was incubated without (A) or with 0.05 µM TbCof (B), negatively stained with uranyl acetate, and observed by electron microscopy. Actin alone maintains long filaments. While only short filaments are observed in the presence of TbCof (B). The scale bars represent 100 nm.

The effect of TbCof on actin filaments was further examined by electron microscopy with preassembled F-actin (Fig. 7). Obvious formation of long actin filaments was observed in the control without TbCof (Fig. 7A). In the presence of TbCof, however, only few long actin filaments remained, and short filaments were frequently observed (Fig. 7B). These observations implied that TbCof may play an important role in sequestering actin monomers, severing or depolymerizing actin filaments.

### Localization of TbCof in Procyclic-form Cells

**Figure 8:**
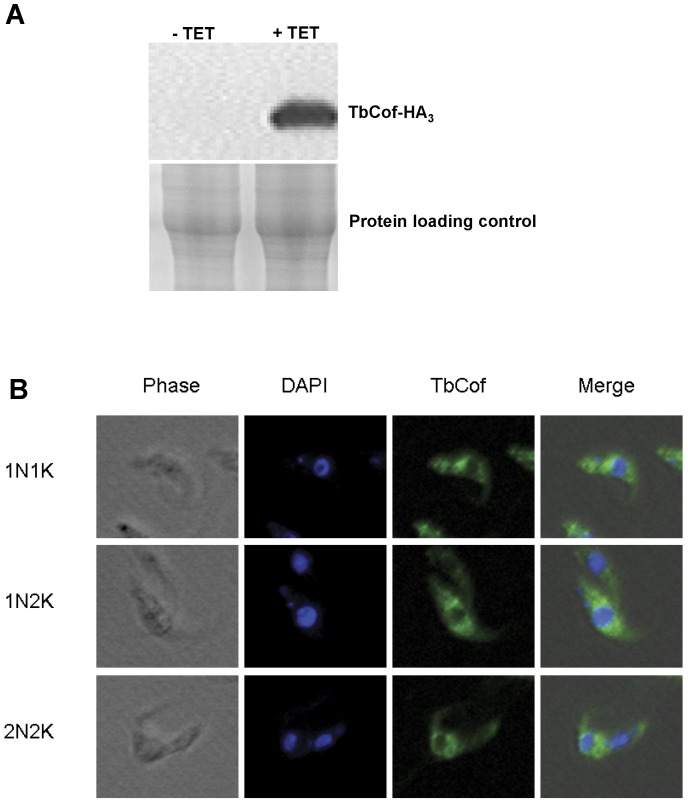
Localization of TbCof in the procylic form *T. brucei*. Cells overexpressing TbCof with an HA_3_-tag at the C-terminus were treated with tetracycline (1 µg/ml) for 2 days. (A) The overexpressed TbCof-HA_3_ examined by western blot using an HA probe. (B) Cells overexpressing TbCof-HA_3_ stained with an HA probe and DAPI, and examined with a fluorescence microscope. TbCof is localized to the cytoplasm throughout the cell cycle.

TbCof-HA_3_ was overexpressed in the procyclic form of *T. brucei*. Western blot indicated a successful expression of TbCof-HA_3_ (Fig. 8A). The localization of TbCof-HA_3_ was then analyzed by fluorescence microscopy. The result showed that TbCof-HA_3_, similar to actin, was localized to the cytoplasm throughout the cell cycle (Fig. 8B), indicating its major role in the cytoplasm.

## Discussion

Actin in *T. brucei*, an early branched organism, has been suggested to be involved in many intracellular processes. However, the regulation of actin filaments in *T. brucei* is poorly understood yet. In this study, our results indicate that *T. brucei* possesses a structurally and functionally conserved ADF/cofilin homologue.

ADF/cofilin family members bind actin monomers and depolymerize actin filaments by either severing filaments or increasing the off-rate at the pointed end [9], [32]. Here, the abilities of G-actin binding and F-actin depolymerization of TbCof are confirmed. In a chemical shift perturbation assay, the extensive loss of peak intensity after the addition of G-actin also indicates interactions between TbCof and G-actin (Fig. S1). The dissociation constant of TbCof interacting with ADP-G-actin derived from ITC (K_d_∼0.08 µM) is similar to those of other ADF/cofilin members, such as LdCof (K_d_∼0.20 µM) and TgADF (K_d_∼0.02 µM), binding to ADP-G-actin [20], [33]. Under low ionic conditions, the binding affinity of TbCof to ADP-G-actin (K_d_∼0.08 µM) is almost five times higher than that of TbCof to ATP-G-actin (K_d_∼0.36 µM). The preference of TbCof for ADP-G-actin is similar to that of the ADF/cofilin members from other organisms [9], [26].

EM and F-actin co-sedimentation assays indicate that TbCof plays an important role in depolymerizing F-actin but does not co-sediment with F-actin, similar to ADF/cofilin from *Caenorhabditis elegans* [34]. This might be explained by the strong ADP-G-actin binding ability of TbCof, which results in its transient interaction with F-actin and the sequestering of ADP-G-actin from the pointed ends. In addition, the ability of F-actin depolymerization of TbCof is pH independent, which is similar to that of ADF/cofilin from *Leishmania donovani* [35]. Moreover, the depletion of TbCof in procyclic-form *T. brucei* does not affect the cell growth (Fig. S2). TbCof is localized throughout the cytoplasm. Our accumulated data indicate a functionally conserved ADF/cofilin in *Trypanosoma brucei*.

ADF/cofilin family members share a highly conserved structure. As expected, TbCof adopts the typical ADF/cofilin fold. Two distinct regions in ADF/cofilin proteins are responsible for recognizing G/F-actin, which have, thus, been named as the G/F-site and the F-site [23], [27], [29]. The G/F-site is required for ADF/cofilin to bind to both G-actin and F-actin, while the F-site is only involved in F-actin binding. Sequence comparisons and the model of TbCof in complex with G-actin demonstrate that TbCof has a conserved G-actin binding site, including the N terminus, the long kinked helix and the turn connecting strand β6 and the C-terminal helix. The F-site of TbCof is comprised of the F-loop between β4 and β5 and the C-terminus of the protein. The conserved residues involved in F-actin binding are present in TbCof. For example, R77 and K79 in the F-loop of TbCof, corresponding to R80 and K82 of yeast cofilin; R130 and R136 in the C-terminal helix of TbCof, corresponding to R135 and R138 of yeast cofilin. All of the residues R80, K82, R135 and R138 of yeast cofilin are essential for F-actin binding [29].

In summary, we have characterized the first ADF/cofilin from *T. brucei* (TbCof) in structure and function. TbCof adopts the conserved ADF/cofilin fold with a central β-sheet surrounded by five α-helices, binds to G-actin, and promotes F-actin depolymerization.

## Materials and Methods

### Protein Preparation

The recombinant TbCof contains 144 residues with an additional N-terminal tag of six histidine residues from residue M1 to H8. The recombinant TbCof was expressed and purified to homogeneity as described previously [15], and then dialyzed into buffer containing 25 mM NaH_2_PO_4_, 100 mM NaCl, 2 mM EDTA, pH 6.8, and concentrated to 8 mg/ml. The final NMR sample contained 0.5 mM recombinant TbCof and 25 mM phosphate, 100 mM NaCl and 2 mM EDTA in 10%:90% D_2_O:H_2_O, pH 6.8.

The proteins used for detecting interactions between TbCof and G/F-actin were purified as His_6_-tagged TbCof fusion proteins with a TEV protease cleavage sequence between the sequences of His_6_-tag and TbCof. After purified with Ni-NTA resin filled column (QIAGEN), the His_6_ tag was cleaved using TEV protease and was removed with Ni-NTA resin filled column, while the TbCof was further purified via gel filtration using a superdex 75 column (Amersham). Protein concentrations were determined using Bradford Reagent (Sangon).

### NMR Spectroscopy, Data Processing and Structure Calculation

The following spectra were recorded on a Bruker DMX600 spectrometer: two-dimensional ^1^H-^15^N HSQC, three-dimensional HNCO, HN(CA)CO, CBCA(CO)NH, CBCANH, H(CC)ONH, HBHA(CO)NH, HC(CO)NH, ^15^N-edited NOESY with mixing times of 100 ms and ^13^C-edited NOESY with mixing 130 ms. The slowly exchanging amides were obtained from a series of two-dimensional^ 1^H-^15^N HSQC spectra. The ^15^N-labeled sample was lyophilized and dissolved in 99.96% D_2_O for exchanging spectra. NMRPipe, NMRDraw [36] and Sparky 3 [37] running on a Linux system were used for NMR data processing and analysis.

The NMR distance restraints for structure calculation were obtained from 3D ^15^N-edited and ^13^C-edited NOESY spectra. Backbone torsion angle restraints were predicted from chemical shifts of five types of nuclei: ^13^Cα, ^13^Cβ, ^13^CO, ^1^Hα, and ^15^NH by using TALOS+ [38]. Hydrogen bond restraints were obtained from the amide protons with slow-exchange. Hydrogen bond restraints were 2.0 Å and 3.0 Å for H-O and N-O, respectively. The program CYANA 3.0 [39] was used for structure calculation. There were totally 200 conformers calculated independently, and 20 lowest-energy structures were selected and analyzed.

MOLMOL [40] and PYMOL [41] were used for analyzing and generating figures for structures. PSVS 1.4 (http://psvs-1_4-dev.nesg.org/) was used to analyze the quality of the structure.

### Isothermal Titration Calorimetry (ITC)

ITC experiments were carried out at 20°C on an ITC 200 calorimeter from MicroCal™ (Northampton, MA, USA). TbCof and the rabbit muscle G-actin (Worthington) were dialyzed in ADP-G-buffer (10 mM Tris, 0.2 mM CaCl_2_, 0.2 mM ADP, 2.0 mM BME, pH 7.4) or ATP-G-buffer (10 mM Tris, 0.2 mM CaCl_2_, 0.2 mM ATP, 2.0 mM BME, pH 7.4). Both of the two proteins were dialyzed at 4°C against 1 liter of buffer. The buffer was changed four times during a 36-hour period to assure that the buffer for both proteins was the same. All the samples were centrifuged at 4°C, 12000 g for 15 min and then degassed for 20 min. The buffer was pretreated using vacuum filtrating and degassing for 20 min before the ITC experiment. The sample cell was filled with 200 µl G-actin as titrant and titrated against TbCof, which was filled in the syringe of 40 µl. The concentrations of ADP-G-actin and ATP-G-actin were 0.015 mM and 0.028 mM, respectively. TbCof was titrated at the concentrations of 0.15 mM and 0.28 mM for ADP/ATP-G-actin, respectively. The injections were performed using a volume of 2 µl per injection, 4 s for the duration of the injection, and with a 120-s interval between the injections. During the titration, the reaction mixture was continuously stirred at 1000 rpm. Control experiments were carried out by injecting TbCof into ADP or ATP G-buffer under the same conditions as TbCof/G-actin titration, to take the heats of dilution and viscous mixing into account. The heats of injection of the control experiment were subtracted from the raw data of G-actin and TbCof titration. The ITC data were analyzed using the ORIGIN version 7.0 software provided by MicroCal™. The heats of binding were normalized with respect to the titrant concentration, and a volume correction was performed to take into account dilution of titrant during each injection. The amount of heat produced per injection was calculated by integration of the area under each peak using a baseline selected by the ORIGIN program, assuming a one site binding model. The dissociation constant (K_d_) and molar enthalpy (ΔH) for the binding of TbCof to actin were determined by non-linear least square fitting to the data.

### HADDOCK Modeling of the TbCof-G-actin

Models of TbCof-G-actin were generated using the HADDOCK webserver [28]. The crystal structure of the C-terminal ADF-H domain of twinfilin (Twf-C) in complex with an actin monomer (PDB ID: 3DAW) [27] was taken as a template to build a model of TbCof-G-actin. Active residues included S4, R93, K95, M96, L97, S99, G114 and Q116 of TbCof and Y143–G146, E167, I341, G342, I345, S350, T351, F352 of actin. Passive residues included M1–M3, P92, T94, Y98, S100, I115 and A117 of TbCof and R147, W340, L346 and L349 of actin. All these residues are in the conserved binding sites between ADF/cofilin and G-actin.

### Actin Filament Sedimentation Assays

Rabbit skeletal muscle actin was incubated in F-buffer (100 mM KCl, 2 mM MgCl_2_, 1 mM ATP and 10 mM Tris, pH 7.4) for 30 min at the concentration of 5 µM to obtain preassembled F-actin. 0, 1, 2.5, 5, 10, or 20 µM TbCof in G-buffer (10 mM Tris, 0.2 mM CaCl_2_, 0.2 mM ATP and 2.0 mM DTT, pH 7.4) was mixed with 3 ml of the polymerized actin filaments and incubated for 30 min. Reactions were then centrifuged in a Beckman Optima MAX Ultracentrifuge in a TLA 100.3 rotor at 75,000 rpm for 1.5h. The supernatants and the pellets were adjusted to the same volume and analyzed by 12% SDS-PAGE. The proteins were visualized by staining with Coomassie Brilliant Blue-R250. All steps were carried out at room temperature.

In order to analyze the effects of pH on the actin binding and depolymerization activities of TbCof, F-actin was incubated with TbCof for 30 min at room temperature in a buffer containing 100 mM KCl, 2 mM MgCl_2_, 1 mM ATP, together with 20 mM 4-(2-Hydroxyethyl)-1-piperazineethanesulfonic acid (pH 6.0, 6.5 and 7.5) or 20 mM Tris-HCl (pH 8.5 and 9.0). The mixtures were centrifuged at 75,000 rpm for 1.5 h, as described above. The supernatant and pellet fractions were analyzed by 12% SDS-PAGE.

### Electron Microscopy

Rabbit skeletal muscle actin was polymerized at a concentration of 5 µM in F-buffer (100 mM KCl, 2 mM MgCl_2_, 1 mM ATP and 10 mM Tris, pH 7.5) for 30 min at room temperature. 0.05 µM TbCof in G-buffer (10 mM Tris, 0.2 mM CaCl_2_, 0.2 mM ATP and 2.0 mM DTT, pH 7.4) was mixed with F-actin for 5 min. After the incubation, samples were fixed on carbon-supported Formvar-coated grids and negatively stained with an aqueous solution of 1% uranyl acetate. Micrographs were taken on a FEI Tecnai F20 TEM transmission electron microscope at 200 kV.

### Cell Culture

Cunningham’s medium [42] supplemented with 15% fetal bovine serum (Hyclone) was used to cultivate the procyclic-form *T. brucei* strain 29–13 [43] at 26°C. Hygromycin B (50 µg/ml) and G418 (15 µg/ml) were added to culture medium to maintain the tetracycline-repressor gene constructs and T7 RNA polymerase in the cells. Culture medium with additional phleomycin (2.5 µg/ml) was used to select transfectants and stabilize the cell line [44].

### Overexpression of TbCof in Procyclic-form *T. brucei*

The full-length gene of TbCof was amplified by PCR, using the following primers: TbCof-OE-F, 5′-AAGCTTGGAATTCCTTTGTGTTACATTCTTGAATG- GCCATGTCTGGTGTTTC-3′; TbCof-OE-R, 5′-CTCGAGCCGGTTCGACTTCAC- TTTGC-3′ (the HindIII and XhoI sites are underlined; 5′-UTR sequence is from 7 bp to 32 bp). The PCR products were purified and subsequently cloned into pLEW100 [43].

The vector pLEW100 containing the full-length gene of TbCof was linearized with NotI for integration into the *T. brucei* rDNA spacer region. Cells overexpressing TbCof were prepared as described before [44]–[46]. The C-terminus of intact TbCof was tagged with a triple hemagglutinin tag (TbCof-HA_3_). Expression of TbCof-HA_3_ was induced by adding 0.1 µg/ml of tetracycline to the medium. After 2 days of induction, cells were harvested and used for western blot and immunofluorescence analyses.

### Western Blot

10^7^ tetracycline-induced cells were harvested and washed twice with PBS. Cells were lysed with 1×SDS-PAGE loading buffer (50 mM Tris-Cl, pH 6.8; 0.1% bromophenol blue; 2% SDS; 10% glycerol; 100 mM DTT). The samples were fractionated, blotted onto a PVDF membrane (Millipore), and incubated with antibodies (Primary antibody: HA probe, sc-7392, Santa Cruz Biotechnology, mouse monoclonal antibody against internal region of influenza hemagglutinin (HA) protein, used at a 1∶1000 dilution; secondary antibody: goat-anti-mouse IgG-HRP sc-2005, Santa Cruz Biotechnology, conjugated with HRP).

### Immunofluorescence Microscopy

Cells were harvested and washed three times with PBS (136 mM NaCl, 3 mM KCl, 16 mM Na_2_HPO_4_, 3 mM KH_2_PO_4_, 40 mM sucrose and 10 mM glucose, pH 7.6), then settled on slides and fixed for 15 min with 4% PFA at room temperature. After fixation, the slides were washed once and blocked with PBS containing 1% BSA and 0.1% Triton X-100 at room temperature for 60 min. After that, the slides were incubated with the primary antibody, HA probe for HA_3_ tag (sc-7392, Santa Cruz Biotechnology, mouse monoclonal antibody against internal region of influenza hemagglutinin (HA) protein, used at a 1∶100 dilution in PBS with 1% BSA), for 90 min. After washing with PBS for 5 min three times, the slides were incubated with the secondary antibody, FITC conjugated anti-mouse IgG (F-6257, Sigma, used at a 1∶100 dilution in PBS with 1% BSA), for 60 min. Finally, slides were washed with PBS and mounted in vectashield in the presence of 1 µg/ml of DAPI. Slides were observed and analyzed on an Olympus phase-contrast and fluorescence microscope.

## Supporting Information

Figure S1 (EPS)

Figure S2 (EPS)

Materials and Methods S1 (DOC)
