# Supplementary material for: Structural and Functional Insight into ADF/Cofilin from Trypanosoma brucei
Source: PLoS One. 2013 Jan 9;8(1):e53639. doi: 10.1371/journal.pone.0053639 (PMC3541276; doi:10.1371/journal.pone.0053639)
Supplement: Materials and Methods S1 — (DOC) [file pone.0053639.s003.doc]

**Supplementary Materials and Methods**

*Chemical shift perturbation*

To identify the interactions between TbCof and G-actin, 0.5 mM ^15^N-labeled TbCof was titrated with unlabeled G-actin to different molar ratios (TbCof/G-actin, 1:0, 1:0.3, 1:0.5 and 1:1). A series of ^1^H-^15^N HSQC spectra of TbCof with or without G-actin were recorded in HEPES buffer (2.0 mM HEPES, 0.2 mM CaCl_2_, 0.2 mM ADP, 2.0 mM BME, pH 7.4). G-actin dissolved in HEPES buffer was titrated stepwise with a sample dilution of less than 10%.

*Construction of stable RNA interference cell line*

Primers used to amplify partial cDNA fragment (264 bp) of TbCof were as follows: TbCof-forward, 5’- CACA CTCGAG TTATGCACATCGTTGATC- 3’; TbCof-reverse, 5’- TCAG AAGCTT TCCCTGGAAACCCTG- 3’ (Hind III site and Xho I site are underlined). PCR products were purified, cloned into pZJM vector with XhoI and HindIII. pZJM vector includes two opposing tetracycline-inducible T7 promoters, which can be used to synthesize double-strand RNA to induce RNA interference in vivo .

pZJM vectors containing the segment of TbCof were linearized with NotI and then transfected into strain 29-13 cells by electroporation. 10^8^ cells were harvested, washed twice with cytomix buffer (120 mM KCl, 0.15 mM CaCl_2_, 10 mM K_2_HPO_4_/KH_2_PO_4_, 25 mM HEPES, 2 mM EGTA, 5 mM MgCl_2_, 2 mM ATP, 5 mM glutathione, and pH adjusted to 7.6 with KOH) and then suspended in 0.45 ml of cytomix buffer containing 25 μg of the linearized constructs. Electroporation was carried out in a 2-mm cuvette (Bio-Rad) using the Gene Pulser (Bio-Rad) with parameters set as follows: 0 Ω resistance, 1.6 kV voltages and 25 μF capacitance. The transfected cells were immediately transferred into 10 ml of fresh Cunningham's medium, and phleomycin (2.5 μg/ml) was added 24 h after transfection. The transfectants were selected under 2.5 μg/ml phleomycin with individual cells cloned by limiting dilutions. The stable transfectants thus selected were grown in the culture medium containing phleomycin . To investigate the effect on cell proliferation after depletion of TbCof, cell numbers were counted at different time points for three times using a hemocytometer.

*Semi-quantitative RT-PCR*

Total RNA was extracted from cells using TRIzol reagent (Sangon). 1 μg of total RNA was used in each reverse transcription reaction. Complementary DNA was synthesized with AMV reverse tran-scriptase (Promega). The reaction buffer was first incubated at 42 °C for 60 min and then 75 °C for 5 min. Primer sets used for evaluating efficiency of RNA interference were as follows: TbCof-RT-forward, 5’- CCCTCGTGACAAATTAATTCTCATCAG- 3’; TbCof-RT-reverse, 5’- TTACCG GTTCGACTTCACTTTGCGGGAG- 3’. 5μl of reverse transcripts was used for PCR and the PCR cycle was maintained at 95 °C for 30 s, 55 °C for 40 s, and 72 °C for 45 s for a total of 25 cycles. α-tubulin mRNA was also analyzed as a sampling control.

**References**

1. Wang Z, Morris JC, Drew ME, Englund PT (2000) Inhibition of Trypanosoma brucei gene expression by RNA interference using an integratable vector with opposing T7 promoters. Journal of Biological Chemistry 275: 40174-40179.

2. Tu X, Kumar P, Li Z, Wang CC (2006) An aurora kinase homologue is involved in regulating both mitosis and cytokinesis in Trypanosoma brucei. Journal of Biological Chemistry 281: 9677-9687.

3. Van den Hoff M, Moorman A, Lamers WH (1992) Electroporation in'intracellular'buffer increases cell survival. Nucleic acids research 20: 2902.

**Supplementary Figure legends**

**Figure S1.** Interactions between TbCof and ADP-G-actin revealed by chemical shift perturbation. To identify the interactions between TbCof and G-actin, 0.5 mM ^15^N-labeled TbCof was titrated with unlabeled G-actin to different molar ratios (TbCof/G-actin 1:0, left top; 1:0.3, right top; 1:0.5, left bottom and 1:1, right bottom). A series of ^1^H-^15^N HSQC spectra of TbCof with or without G-actin were recorded in HEPES buffer (2.0 mM HEPES, 0.2 mM CaCl_2_, 0.2 mM ADP, 2.0 mM BME, pH 7.4). G-actin was titrated stepwise with a sample dilution of less than 10%.

**Figure S2.** Effects of TbCof knockdown on the procyclic-form *T. brucei*. Tetracycline-inducible TbCof-deficient cell line were incubated at 26 °C in Cunningham's medium without (-TET, as control) or with (+TET, as induction) 2 μg/ml of tetracycline. Cell numbers were counted daily and tabulated. The inset shows the intracellular level of TbCof mRNA after 3 days of induction as monitored by semiquantitative RT-PCR. Level of α-tubulin (α-TUB) mRNA was analyzed as sampling control.
